# Supplementary material for: Inhibition of Clinical MRSA Isolates by Coagulase Negative Staphylococci of Human Origin
Source: Antibiotics (Basel). 2024 Apr 8;13(4):338. doi: 10.3390/antibiotics13040338 (PMC11047365; doi:10.3390/antibiotics13040338)
Supplement: Supplementary file 1 [file antibiotics-13-00338-s001.zip › antibiotics-2926133-supplementary.pdf]

Table S.1. Summary of activity observed from isolates shortlisted for whole genome sequencing.

Isolate	Deferred antagonism assay	Biofilm inhibition	Growth inhibition of MRSA M at T12
**C14**	• Inhibition of *M. luteus* and MRSA strains G and R	• Significant inhibition of MRSA, V, R, M, and E biofilm formation	Yes
**C33**	• Inhibition of *M. luteus*	• Significant inhibition of MRSA, V, R, M, and T biofilm formation	Yes
**E4**	• Inhibition of *M. luteus*	• Significant inhibition of MRSA, V, R, M, and E biofilm formation	Yes
**E6**	• Inhibition of *M. luteus*	• Significant inhibition of MRSA, R, M, T, and E biofilm formation	Yes
**E54**	• Inhibition of *M. luteus* and MRSA E	• Significant inhibition of MRSA, R, M, and E biofilm formation	Yes
**E67**	• Inhibition of *M. luteus* and MRSA strains C, D, E, G, H, I, K, L, M, N, P, R, T, W, U and V.	• Significant inhibition of MRSA, V, R, M, T, and E biofilm formation	Yes
**E89**	• Inhibition of *M. luteus*	• Significant inhibition of MRSA R, M, T, and E biofilm formation	Yes
**E96**	• Inhibition of *M. luteus*	• Significant inhibition of MRSA R, M, T, and E biofilm formation	Yes
**E99**	• Inhibition of *M. luteus*	• Significant inhibition of MRSA V, R, T, and E biofilm formation	Yes
**E100**	• Inhibition of *M. luteus*	• Significant inhibition of MRSA V, R, T, and E biofilm formation	Yes
**E170**	• Inhibition of *M. luteus* and MRSA W	• Significant inhibition of MRSA R, M, T, and E biofilm formation	Yes

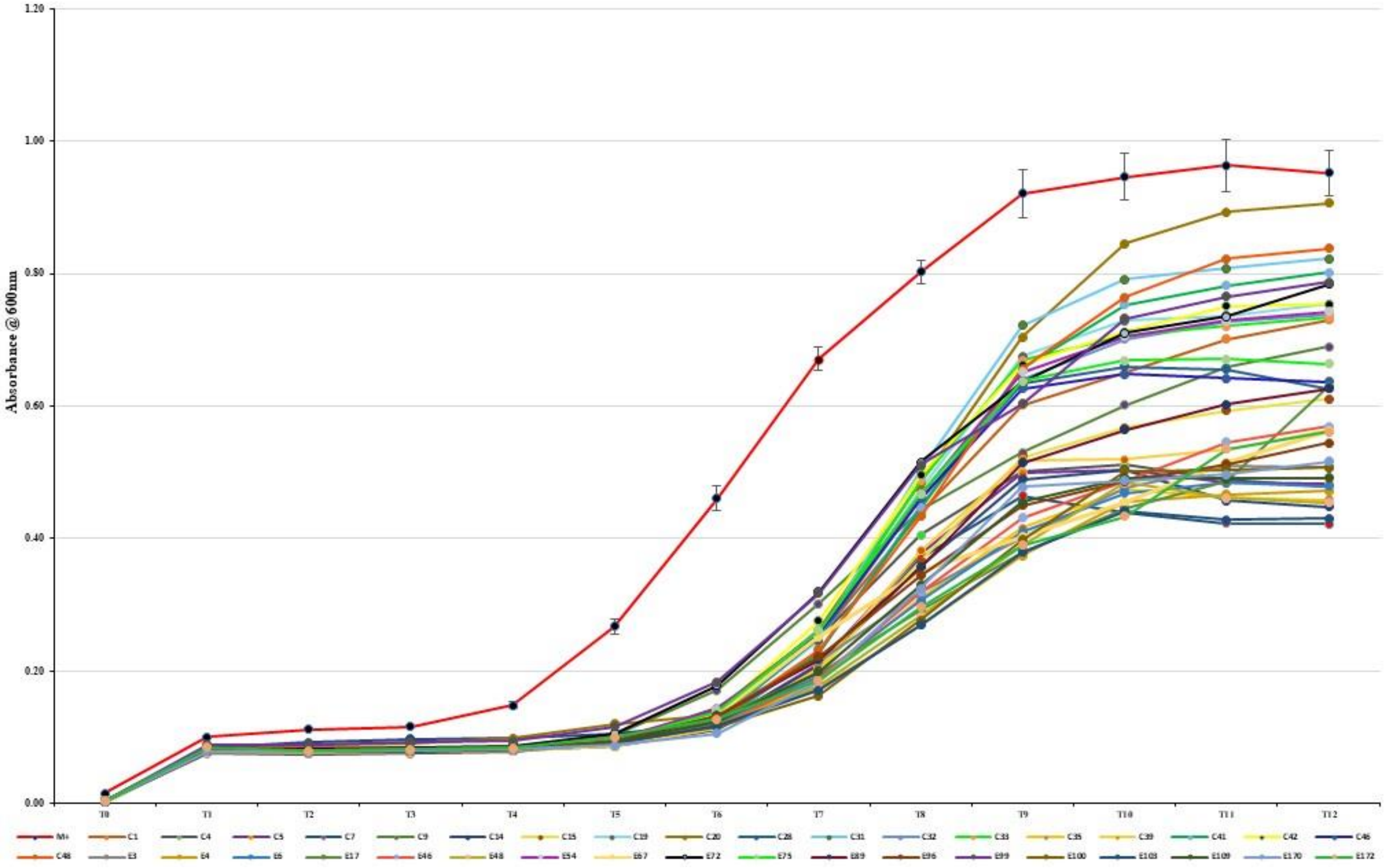

Figure S.1. Hourly absorbance readings of the growth of representative MRSA indicator strain, MRSA M, cultured in the presence of the acid-neutralized CFS prepared from 37 individual CoNS overnight cultures. The cultures were incubated at 37°C with continuous pulsing. Absorbances taken every 60 minutes over a 12-hour period at 600 nm. Plotted points are the average of three biological repeats minus the sample blank (uninoculated broth). A positive control was created by incubating MRSA M (represented by M+ in the figure) in the absence of CFS. Error bars for MRSA cultured in the presence of the CFS were very small and omitted from the figure for clarity.

Table S.2. Genomic information obtained following the sequencing of 11 shortlisted CoNS isolates by Microbes NG (Birmingham, UK). The quality of the sequenced genomes was determined through QUAST and CheckM, and sequence coverage was determined using Bowtie2 and SAMtools.

Isolate	E99	E100	E89	E54	E6	E67	E170	E96	C33	E4	C14
**# contigs (>= 0 bp)**	57	60	59	63	57	67	55	38	63	47	50
**# contigs (>= 1000 bp)**	34	30	25	31	31	31	14	12	31	14	41
**# contigs (>= 5000 bp)**	26	23	20	25	26	23	11	8	25	9	33
**# contigs (>= 10000 bp)**	23	21	19	24	22	22	10	8	21	9	28
**# contigs (>= 25000 bp)**	21	17	17	20	18	19	7	7	18	8	17
**# contigs (>= 50000 bp)**	15	15	15	15	16	15	6	5	13	6	6
**Total length (>= 0 bp)**	2412971	2559883	2409487	2425182	2481282	2522476	2526178	2496969	2497126	2501795	2538711
**Total length (>= 1000 bp)**	2406443	2550413	2399860	2414828	2472909	2512185	2512249	2490011	2489002	2491656	2526700
**Total length (>= 5000 bp)**	2386294	2534389	2392622	2400335	2456720	2489047	2506724	2484166	2472606	2481984	2399831
**Total length (>= 10000 bp)**	2361944	2517992	2386134	2391111	2424377	2480528	2501709	2484166	2442951	2481984	2300217
**Total length (>= 25000 bp)**	2323091	2451900	2357906	2318469	2368133	2432970	2439135	2464006	2407536	2458154	2199981
**Total length (>= 50000 bp)**	2116033	2371329	2270246	2143095	2281372	2283867	2399820	2395255	2209510	2393212	2176311
**# contigs**	35	34	28	35	36	35	18	14	33	16	44
**Largest contig**	284537	465573	490174	280833	328253	443168	726371	1118993	502608	899999	356753
**Total length**	2407414	2553246	2401913	2417951	2476147	2514912	2515531	2491238	2490242	2492844	2273229
**GC (%)**	32	31.92	32.09	32.03	31.99	31.91	32.78	32.83	31.91	32.8	31.37

**N50**	147472	201293	173367	139835	181306	185285	694797	904609	199479	564903	199432
**N90**	39240	51665	75394	40238	55244	51433	141193	116844	47523	134308	50345
**auN**	156013.8	220461.9	212228.9	157055.8	169196.7	217089.9	556877.9	851925.4	244154.6	565060.2	234561.5
**L50**	6	5	5	6	6	5	2	2	4	2	5
**L90**	17	14	14	16	16	15	5	4	14	5	10
**# N's per 100 kbp**	0	0	0	0	0	0	0	0	0	0	0
**Contamination**	0	0	0.37	0	0	0	0.13	0.66	0.1	0.08	0
**heterogeneity**	0	0	50	0	0	0	33.33	0	0	0	0
**coverage**	93.0481	128.955	31.2918	82.7517	127.613	112.873	103.935	101.033	112.492	128.3	128
